# Immunizing Pregnant Women and Infants (IMPRINT) Network: Building and Sustaining an Interdisciplinary Network Tackling the Complex Challenges of Vaccination in Pregnancy and Early Life

**DOI:** 10.1097/INF.0000000000004697

**Published:** 2025-02-14

**Authors:** Beate Kampmann, Christine E. Jones, Hannah Faber

**Affiliations:** * Faculty of Infectious Diseases, The London School of Hygiene and Tropical Medicine, London, UK; † Charité Centre for Global Health; Charité Universitätsmedizin, Berlin, Germany; ‡ Faculty of Medicine and Institute for Life Sciences, University of Southampton and NIHR Southampton Clinical Research Facility and NIHR Southampton Biomedical Research Centre, University Hospital Southampton NHS Foundation Trust; § LINQ management GmbH, Berlin, Germany

**Keywords:** vaccines, pregnancy, global

## Abstract

IMPRINT is a UK-funded, international research network that supports interdisciplinary research addressing key challenges in the best use of vaccines in pregnancy and in newborns. The goal of this short review paper is to introduce readers to the network’s aims and objectives and highlight its initiatives and outputs for research and innovation within the UK and in low- and middle-income countries. The long-term goal of this network is to contribute to improved maternal and newborn health globally through the safe and effective use of vaccines.

## Introduction

Despite significant progress, mortality in children under the age of five remains a global problem, with nearly 50 percent of deaths now occurring in the neonatal period. These are frequently related to infectious diseases that could be prevented by vaccination, such as pertussis, respiratory syncytial virus (RSV) or tetanus. In addition, several infections have more severe outcomes in pregnancy, e.g. influenza, Sars-CoV-2. Preventing these infections in pregnant women and their newborns is therefore a logical intervention which relies on transfer of the antigen-specific antibody via the placenta that already occurs in every pregnancy, a “gift of nature”. On the back of demonstrated feasibility, safe implementation and demonstrated success for the prevention of diseases like tetanus, influenza, and whooping cough and with novel vaccines on the horizon, the IMPRINT network was established in 2017 as one of the five UKRI-funded vaccine-focused networks with a global reach. https://www.ukri.org/news/global-vaccine-networks-tackling-infections-around-the-world/

### History and organization of IMPRINT

Since 2017, the Immunising Pregnant Women and Infants (IMPRINT) Network (https://www.imprint-network.co.uk) focuses on maternal and neonatal immunization. The network is coordinated by Network Director Prof. Beate Kampmann (London School of Hygiene and Tropical Medicine) and Co-Director Dr Chrissie Jones (University of Southampton) and has received over £4.2 million in funding from the UK’s Medical Research Council (MRC), as well as the Global Challenges Research Fund (GCRF) and Biotechnology and Biological Sciences Research Council (BBSRC). The network is directed by an international steering committee (https://www.imprint-network.co.uk/people) and its management facilitated by LINQ MANAGEMENT, BERLIN (https://linq-management.com).

IMPRINT supports interdisciplinary research that addresses key challenges in the best use of vaccines in pregnancy and in newborns. The network’s goal is to strengthen capacity for research and innovation within the UK and in low- and middle-income countries, and in the long term, to improve maternal and newborn health globally. Through collaborative research projects, members seek to answer e.g. fundamental biological questions about antibody transfer across the placenta, how to induce the “best” antibody, and study its effect on the maturing newborn immune system. Members also investigate how co-factors such as nutrition or other infections might change the way in which mothers and infants are protected by vaccines. Additionally, the IMPRINT network seeks to find out why vaccine hesitancy is higher amongst women in some countries as opposed to others, and how vaccine safety and effectiveness can be best monitored in a world where different countries’ health care systems function very differently.

### Progress since inception

IMPRINT has established a sustainable network of stakeholders from basic science, immunology, vaccinology, social sciences, industry, public health, and national and international policy makers (https://www.imprint-network.co.uk/membership). With such a multidisciplinary set of skills and experiences represented in the network, IMPRINT is well situated to address the complex challenges of maternal and neonatal immunization. The diversity of the network is further reflected in its international membership: of the 54 countries represented in IMPRINT, 34 are low-and middle-income countries and 20 are high-income countries. Over the past seven years, the network has grown to include more than 400 members, 39% of whom are from low- and middle-income countries and 61% from high-income countries. Women represent 60% of all network members, men represent 40%.

IMPRINT continuously strives to strengthen its network and to create opportunities for its members to expand their knowledge and exchange ideas. Since 2017, IMPRINT has organized several meetings for members to reflect on the current state of maternal immunization research and shape the agenda for future collaboration across the network. Past meetings have included network meetings in the UK (2018, 2021 and 2023) and Belgium (2017), as well as dedicated IMPRINT sessions at the International Maternal and Neonatal Immunisation Symposium (INMIS) in Canada in 2019 and Costa Rica in 2024. IMPRINT was also able to foster collaboration and continued building its network throughout the COVID-19 pandemic, during which members met virtually to share their work and maintain their research efforts into effective and safe vaccines for mothers and infants.

To support its members in their important work, in the past seven years IMPRINT has offered a webinar series designed to expand members’ skillsets and expand their expertise. IMPRINT has also supported 12 pump-priming projects, 8 progression projects, 4 career fellowships and 10 public engagement projects have been awarded funding through the network. Through these collaborative projects and fellowships, IMPRINT members can address complex issues concerning vaccines for mothers and infants on a vast scale. Particularly through public engagement projects, members endeavor to reach new, global, and diverse audiences to raise public awareness through imaginative, high-quality, and inclusive public engagement in the UK and IMPRINT’s partner countries.

Additionally, the network has facilitated a few training opportunities, including media and public engagement training, and has made funds available for members to attend training offered by other organization and initiatives working in the fields of immunology and vaccinology.

### Scientific achievements

IMPRINT has supported members and collaborators by funding projects three focused research areas which are instrumental to advance the field of maternal and neonatal immunization and lead towards our shared objectives: these address biological challenges, implementation obstacles and advocacy initiatives.

Key scientific findings addressing biological challenges relate to mechanism of antibody transfer across the placenta, timing of maternal vaccination and antibody levels, factors affecting antibody transfer efficiency, impact on neonatal immune development, limitations, and duration of protection.

Implementation challenges have been tackled through research in the areas of perceived safety and efficacy of vaccines, trust in healthcare provider recommendations, cultural beliefs and social norms, access and availability of vaccination services, development of pregnancy registries and integration of vaccination within routine antenatal care platforms.

During the COVID-19 pandemic, IMPRINT contributed to the COVAX Maternal Immunisation group who met regularly to develop protocols for safe use of COVID-19 vaccines in pregnancy. This work continues to inform the WHO and CEPI guidelines.

Details of these programs of work as well as their output are publicly available on the IMPRINT website (https://www.imprint-network.co.uk/projects).

The network’s advocacy efforts have continuously addressed gaps in knowledge and awareness through public engagement activities, strengthening healthcare provider communication, advocacy and development of culturally tailored public health campaigns and culturally sensitive research practices. An inspiring example of this activity is our arts project “Clothes with protection” which commissioned ten fashion designers from Uganda to create clothing that highlights the importance of vaccination in pregnancy in Africa. A short film and exhibition materials are taking this project further afield and showcase the creativity of the IMPRINT community, not only in science but also in public engagement. https://www.imprint-network.co.uk/videos.

To ensure that projects continue to have an impact even after they have been completed, IMPRINT has also created a Public Engagement Toolkit for exclusive use of its members. The toolkit provides summaries of past public engagement projects, as well as case studies and a content library which features videos, radio messages and flyers created within the projects. Through the toolkit, members have shared key lessons and best practices to enable all IMPRINT members to leverage the knowledge gained in public engagement projects in their future work.

Publicly available resources for health care workers and pregnant women alike have been developed and are accessible via the website (https://www.imprint-network.co.uk/videos).

These resources can be shared on request and with acknowledgement of IMPRINT and its funders.

Additionally, IMPRINT has created opportunities for its members to engage with industry partners, for example through funding an industry placement in collaboration with Pfizer.

## IMPRINT at INMIS

To showcase the recent output from the network, a dedicated session was held at the 6^th^ International Neonatal and Maternal Immunization Symposium (INMIS 2024) meeting in Costa Rica, where members of the network were able to present their accepted abstracts in short talks. Travel awards enabled the network to sponsor many young researchers from resource-poorer countries.

Given IMPRINT’s focus on the conduct of clinical trials of vaccines given in pregnancy, the first session was dedicated to updates from recent trials using pertussis and RSV vaccines. The data presented addressed aspects of biological challenges by presenting set-up and immunogenicity data of pertussis studies in Uganda and The Gambia, as well as acceptability and access issues to the new RSV vaccines in Europe. A large collaborative study between many members of the IMPRINT network was also presented, examining seroprevalence and transplacental transfer of antibody against measles.

The second session addressed challenges and implementation of pregnancy registries in resource-poorer settings. This topic is becoming more and more urgent, given the need to acquire background data on pregnancy and neonatal outcomes prior to the introduction of new vaccines, such as respiratory syncytial virus and hopefully group B streptococcus in due course. Different approaches from streamlining parameters to be collected via unified protocols to electronic solutions and overarching safety reporting platforms were presented.

Most details of these presentations are contained as publications in this Special Issue reporting from INMIS 2024 and are identified as IMPRINT-funded projects.

### Going forward

The meeting in Costa Rica concluded with the announcements of further funding opportunities via IMPRINT in the shape of visiting scientist fellowships, possible industry placements and a further round of the very popular public engagement grants (https://www.imprint-network.co.uk/funding).

Interested parties can contact IMPRINT via the website and join the network via a simple application process. (https://www.imprint-network.co.uk/membership)

